# Supplementary material for: Curated and harmonised transcriptomics datasets of interstitial lung diseases
Source: Data Brief. 2025 Oct 14;63:112139. doi: 10.1016/j.dib.2025.112139 (PMC12581653; doi:10.1016/j.dib.2025.112139)
Supplement: Supplementary file 1 [file mmc1.zip › Supplementary_material/RNA-seq/fastQC reports/GSE184316/TRIMMED_SRR15926778_fastqc.html]

TRIMMED\_SRR15926778.fastq.gz FastQC Report 

FastQC Report

Sat 3 Dec 2022  
TRIMMED\_SRR15926778.fastq.gz

## Summary

- Basic Statistics
- Per base sequence quality
- Per sequence quality scores
- Per base sequence content
- Per sequence GC content
- Per base N content
- Sequence Length Distribution
- Sequence Duplication Levels
- Overrepresented sequences
- Adapter Content

## Basic Statistics

| Measure | Value |
| --- | --- |
| Filename | TRIMMED\_SRR15926778.fastq.gz |
| File type | Conventional base calls |
| Encoding | Sanger / Illumina 1.9 |
| Total Sequences | 7565333 |
| Sequences flagged as poor quality | 0 |
| Sequence length | 35-371 |
| %GC | 51 |

## Per base sequence quality

## Per sequence quality scores

## Per base sequence content

## Per sequence GC content

## Per base N content

## Sequence Length Distribution

## Sequence Duplication Levels

## Overrepresented sequences

| Sequence | Count | Percentage | Possible Source |
| --- | --- | --- | --- |
| CAGTGGGGCCACGAGCTGAGTGCGTCCTGTCACTCCACTCCCATGTCCCT | 115594 | 1.52794331723402 | No Hit |
| AGGCTACACACTGAGGACTCTGTTCCTCCCCTTTCCGCCTAGGGGAAAGT | 108444 | 1.4334332672467953 | No Hit |
| CACCTCCCCGAAGCTCAGGGAGAGCCCTGTTAGGGCCGCCTCTGGCCCTA | 104971 | 1.387526497511742 | No Hit |
| GGGAAGCGGGGAATGTCTACGTGCGTATGCACGTGGCACTCTCTGCCCGA | 87267 | 1.1535116828300882 | No Hit |
| CAATCCACTTGATCCCAACTCATCTCTCATTTATTTCGGCTTCTTTTATT | 74785 | 0.988522250111132 | No Hit |
| TTACAAAAGCAGAATAAAAGCGAAAAGAAATGAAAATGTTACACTACATT | 71147 | 0.9404344792225273 | No Hit |
| ACCTCTCTCTGGGCTTCTATTTCGACCGCGATGATGTGGCTCTGGAAGGC | 38982 | 0.5152714361681106 | No Hit |
| CCCTGGCTGCTACAGGAGAATAGCAGACAGGTATAGTTAAGAAACAACTT | 36948 | 0.4883856401297867 | No Hit |
| AGCTGCTAAGTGCTGTGTTGTCGTTCCCCCTGCTTAAAATAAAGTTGTTT | 32692 | 0.43212902855697166 | No Hit |
| TGATATGGTTCCTGGCAAGCCCATGTGTGTTGAGAGCTTCTCAGACTATC | 32427 | 0.4286262085224801 | No Hit |
| TGATGACACCCACCGCAACTGTCTGTCTCATATCACGAACAGCAAAGCGA | 29018 | 0.38356540287122853 | No Hit |
| TCAGGAGACGCTCGTAGCCCTCGCGCTTCTCCTCGGCCAATTCGCGGAAG | 23154 | 0.30605394369289496 | No Hit |
| CAGCAGTTTACACCTGCTAGGGTGTTCAAAGGTCAGTGCTATAGAAATTC | 22982 | 0.3037804152176778 | No Hit |
| TCAGGAGACGCTCGTAGCCCTCGCGCTTCTCCTCGGCCAGTTCGCGGAAG | 22622 | 0.29902186724629304 | No Hit |
| AACAAGCACAAAGCCAAGAAAACCAACGATGCCAGATACTGAATTTCTAT | 22123 | 0.2924259910304014 | No Hit |
| GGCATCAAAAGTGATGGACTGCCCATTGCTGGAGAAGACCTTCTCTCCTA | 20545 | 0.271567689089165 | No Hit |
| TACCACTGCCACCTTCTCCATCGGCTCCACTGGCCTCGTGGTGTATGACT | 19276 | 0.25479380749003383 | No Hit |
| AGACATCAAATCCTGCAGACAAGGGGAGCCCTCAGTCTGCAGGGCTCCAT | 17720 | 0.23422630570260425 | No Hit |
| CTTCATGATGTAGCAGCAGGTGCCAGGGGCTGGCTTGTAGGCGATCAGCA | 17664 | 0.23348608712927774 | No Hit |
| GGCAATGAGCGGTTCCGCTGCCCTGAGGCACTCTTCCAGCCTTCCTTCCT | 16589 | 0.2192765341591705 | No Hit |
| GTCCACGTCACACTTCATGATGGAGTTGAAGGTAGTTTCGTGGATGCCAC | 15430 | 0.20395665332907353 | No Hit |
| CGATCAGCACGGGCTCGTACTCTGCCACAAACTGATCACACTGCTTCTGG | 12119 | 0.16019123018114337 | No Hit |
| ACGTTTACCTGTCCATGTCTTACTACTTTGACCGCGATGATGTGGCTTTG | 12047 | 0.15923952058686644 | No Hit |
| CAGCATGTTCCCTCTCCTCATGAGATTGGTGAAGAAAGTATTTGGCAAAG | 11771 | 0.15559130047547148 | No Hit |
| CTCTCTTTCTGGCCTGGAGGCTATCCAGCGTACTCCAAAGATTCAGGTTT | 11715 | 0.15485108190214494 | No Hit |
| GAAACCCAGACACATAGCAATTCAGGAAATTTGACTTTCCATTCTCTGCT | 11276 | 0.14904829701481745 | No Hit |
| CAATCCACTTGATCCCAACTCATCTCTCATTATTTCGGCTTCTTTTATTC | 11256 | 0.14878393323862943 | No Hit |
| TCTTGGGTTATAATCATTGGCAATGTTGTAATCAACAACCCATGTTATTT | 11144 | 0.1473034960919764 | No Hit |
| GTCTCAACATCTGTGTTGGGGAGAGTGGAGACAGACTGACGCGAGCAGCC | 10972 | 0.14502996761675924 | No Hit |
| GCCGACAAGACCAACGTCAAGGCCGCCTGGGGTAAGGTCGGCGCGCACGC | 10820 | 0.14302080291773012 | No Hit |
| GGAAAGGGGGCACTGACTGGTGATGCTGATATTGGAGTGCATCTGAGGCT | 10467 | 0.1383547822680112 | No Hit |
| ACAAGAAGTACATCAAAGATTACATGAAATCAATCAAAGGGAAACTTGAA | 10106 | 0.13358301610781706 | No Hit |
| GGAGAAAAACAGCACCAAGCAGGAGATCCTGGCTGCTCTTGAGAAAGGCT | 10032 | 0.13260487013592132 | No Hit |
| CTGACAGTGTATCTAGCTTTGGAAAACACAGGGGTCTGCCCTGTGAGCTG | 9987 | 0.13201005163949822 | No Hit |
| TTGGAGTGGGAAGCGGGGAATGTCTACGTGCGTATGCACGTGGCACTCTC | 9441 | 0.1247929205495647 | No Hit |
| CATTGTGTTGTGCTAAACGCTGGGAGGGTACAAGTTGGTCATTCCTAAAT | 9338 | 0.1234314471021963 | No Hit |
| CAATGGGGTACTTCAGGGTCAGGATGCCACGCTTGCTCTGGGCCTCGTCG | 9287 | 0.12275731947291679 | No Hit |
| CAGTGGGCCACGAGCTGAGTGCGTCCTGTCACTCCACTCCCATGTCCCTT | 9067 | 0.11984931793484835 | No Hit |
| AGGTCTTGGTGGTGGGGAAGGACAGGAACATCCTCTCCAGGGCCTCCGCA | 8914 | 0.11782693504700983 | No Hit |
| CCAAGATCTTCTTGATGTCCTTGGCGCTGGGGGAGGAGTTGCCCCCTAGG | 8843 | 0.11688844364154229 | No Hit |
| ATCAAAGAAGAACATGTGATCATCCAGGCCGAGTTCTATCTGAATCCTGA | 8697 | 0.1149585880753696 | No Hit |
| CAAAATACTGCTTTTACAAAAGCAGAATAAAAGCGAAAAGAAATGAAAAT | 8500 | 0.11235460487991739 | No Hit |
| GGAAGACAGTGTGGCGATTCCTCAGGGATCTAGAACTAGAAATACCATTG | 8223 | 0.10869316657971301 | No Hit |
| GGCAATGAGCGGTTCCGGTGTCCGGAGGCGCTGTTCCAGCCTTCCTTCCT | 8140 | 0.10759605690853266 | No Hit |
| GGATCTTGGCTTTCTCTTTCCTCTTCTCCTCCAGGGTGGCTGTCACTGCC | 8049 | 0.10639320172687707 | No Hit |
| GTCCACGTCACACTTCATGATGGAGTTGAAGGTGGTCTCGTGGATGCCGC | 8029 | 0.10612883795068902 | No Hit |
| CGAAGCGGTTGTACTTGCGGATGTAGTGCAGATAGTCTCGGCGGATGACA | 8026 | 0.10608918338426081 | No Hit |
| AGTAATCTGCAATGCAATCACAATGCCCAAACTAGACCTGCCATTTCTCA | 7698 | 0.10175361745477693 | No Hit |

## Adapter Content

Produced by FastQC (version 0.11.7)
